# Scleral communication between Glaucoma drainage device capsule and the suprachoroidal space simulating amelanotic choroidal melanoma

**DOI:** 10.5935/0004-2749.20230073

**Published:** 2023

**Authors:** Diego Queiroz, Gustavo Porto Lustosa, Diego Mascato, Melina Correia Morales, Arthur Gustavo Fernandes, Rodrigo Antonio Brant Fernandes

**Affiliations:** 1 Ophthal Hospital Especializado Ltda, São Paulo, SP, Brasil.; 2 Departamento de Oftalmologia e Ciências Visuais, Escola Paulista de Medicina, Universidade Federal de São Paulo, São Paulo, SP, Brasil.

**Keywords:** Vitrectomy/adverse effects, Retinal detachment, Glaucoma, Glaucoma drainage implants, Choroid neoplasms, Melanoma, Fluorescein angiography, Dexamethasone, Humans, Case report, Descolamento retiniano, Desprendimento da retina, Glaucoma, Implantes para drenagem de glaucoma, Neoplasias da coroide, Melanoma, Angiofluoresceínografia, Dexametasona, Humanos, Relatos de casos

## Abstract

This is a case report involving a 56-year-old male patient with a history of pars plana vitrectomy due to a rhegmatogenous retinal detachment in the right eye that resulted in the implantation of a drainage device after the patient developed secondary glaucoma. Two years after the device’s implantation, the patient was referred to our care as his visual acuity had decreased to 20/200 (1.00 LogMAR). At the fundus evaluation, a choroidal amelanotic elevation was observed at the upper temporal equator, and a potential diagnosis was made of amelanotic choroidal melanoma. The ultrasound exam visualized the patient’s implanted superotemporal justabulbar drainage device, which revealed a transscleral communication from the plate fibrocapsular’s draining space to the suprachoroidal space (fistula). The ultrasound also revealed a focal pocket of choroidal detachment in the patient’s superotemporal region, simulating an amelanotic choroidal melanoma. A new pars plana vitrectomy was performed to remove the internal limiting membrane without repercussions at the fistula site. The patient’s recovery progressed well, and he regained a visual acuity of 20/70 (0.55 LogMAR). To the best of our knowledge, this is the first case report of this condition.

## Abstract (pt)

Relato de caso de paciente 56 anos, sexo masculino, com histórico de vitrectomia via pars plana por descolamento de retina em olho direito e posterior implante de dispositivo de drenagem por glaucoma secundário. Dois anos após o procedimento foi encaminhado ao serviço por baixa de acuidade visual (AV) de 20/200 (1.00 LogMAR). À fundoscopia, observou-se uma elevação amelanótica temporal no equador com hipótese diagnóstica de melanoma de coroide amelanótico. O exame de ultrassom mostrou implante de dispositivo de drenagem justabulbar temporal superior com comunicação transescleral para espaço subcoroidal (fístula), sugerindo bolsão focal de descolamento de coroide em equador temporal superior simulando melanoma de coroide amelanótico. O paciente foi abordado cirurgicamente devido membrana epirretiniana com nova vitrectomia via pars plana para peeling de membrana limitante interna, sem repercussões no local da fístula, evoluindo bem com acuidade visual de 20/70 (0.55 LogMAR). Ao nosso conhecimento, este é o primeiro caso relatado nessa condição.

## INTRODUCTION

Glaucoma is the leading cause of irreversible blindness worldwide, and intraocular pressure (IOP), which is the disease’s only modifiable risk factor, must be monitored closely in patients in order to prevent vision loss as the disease progresses^(1)^. Surgical treatment of glaucoma is necessary for cases in which clinical treat­ments have failed. Trabeculectomy (TRAB) with the use of antimetabolites is the most widely used filtering surgery in the treatment of glaucoma, although the literature has shown that up to 10% of these procedures fail per year. Certain risk factors, such as having uveitic or neovascular glaucoma or being young or Black, have been reported to be associated with the increased failure rates of TRAB^(2)^.

Recently, the number of TRAB surgeries has been steadily decreasing and has been replaced by the implantation of drainage tubes. The surgical complications of TRAB may explain this decrease^(2)^.

Glaucoma drainage devices (GDD) can lower IOP by promoting the movement of aqueous humor to an extrascleral reservoir through a small caliber tube. The most common indications for GDD implantation are neovascular, refractory (no response to other glaucoma treatment), aphakic, and traumatic glaucoma^(3)^.

Although some studies have shown a lower rate of complications with tube implants compared with trabeculectomies, the potential for adverse outcomes with seton implantations still exists^(4)^. Complications associated with GDD include choroidal effusion, a shallow anterior chamber, diplopia, strabismus, tube-corneal touch, corneal edema, hyphema, hypotony, endophthalmitis, and erosion^(4)^.

The following case report demonstrates an acquired scleral communication between the GDD’s fibrous capsule and the suprachoroidal space after the implantation of an Ahmed GDD. This communication, which was not caused by a hyperfiltration, occurred through a scleral fistula to the suprachoroidal space, simulating an amelanotic choroidal melanoma.

## CASE REPORT

This case report involves a 56-year-old male patient with a history of rhegmatogenous retinal detachment in the right eye, which occurred in 2016. The patient underwent a pars plana vitrectomy (PPV) and phacoemulsification with an intraocular lens implant and an injection of silicone oil to repair his retina. The silicone oil was removed three months after surgery.

After one month, the patient developed increased IOP and secondary glaucoma, which was unmanageable with topical medication, and an Ahmed GDD implantation via the upper temporal pars plana was performed in 2018. After the GDD implantation, the patients’ IOP remained stable and manageable without the use of any topical medications.

During a routine consultation, a lesion was observed at the patient’s upper temporal equator, and the surgeon performed laser around the lesion before referring him to our care. The patient was referred to our department in 2020 with complaints of metamorphopsia and decreased visual acuity in his right eye.

The patient showed a visual acuity of 20/200 in his right eye (1.00 logMAR) and 20/20 in his left eye (0.00 logMAR) upon examination. At biomicroscopy, the right eye presented a pseudophakia with an Ahmed GDD. The GDD’s silicon tube was positioned in the pars plana of the superotemporal region, and the IOP was 18 mmHg, The left eye had no noticeable changes and had an IOP of 17 mmHg.

**Figure 1:**
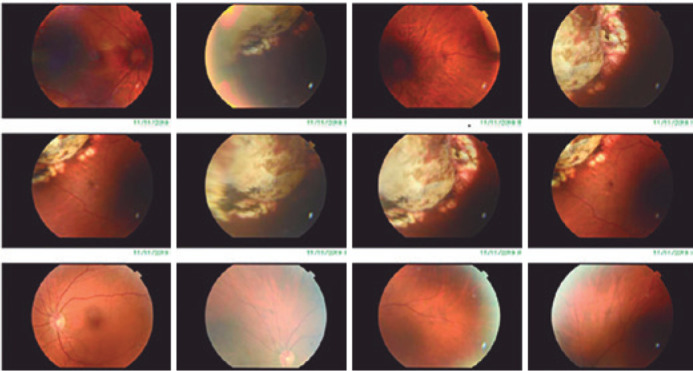
Retinography of the right eye. Elevated amelanotic lesion in the upper temporal equator with laser marks surrounding the lesion (TRC-50DX, Topcon, Japan).

At the fundus evaluation, we observed a choroidal amelanotic elevation (Figure 1) at the upper temporal equator. We also observed that although the patient’s retina was attached, there was an observable epiretinal membrane. None of these observations were present at the patient’s initial retinal evaluation after his referral.

A diagnosis of amelanotic choroidal melanoma was suggested, and an ocular ultrasound and fundus exams (fundus photo, angiofluoresceinography, and ocular coherence tomography [OCT]) were requested.

**Figure 2:**
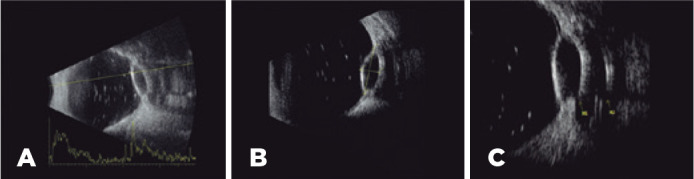
Ultrasonography showing residual emulsified silicon oil in the vitreous cavity, a focal pocket of choroidal detachment, and a GDD juxtaposed at the superotemporal equator (A, longitudinal B-scan). The choroidal detachment was 3.91 mm thick and 9.96 mm in circumferential diameter (B, transverse B-scan). In the transverse view, a transscleral communication (yellow arrow M1) was observed in the inferior aspect of the choroidal detachment, juxtaposed at the inferior aspect of the fibrocapsular space containing the plate (echodense line, yellow arrow M2) of the GDD (C) (Aviso, Quantel Medical, France). GDD, glaucoma drainage device.

The ultrasound exam revealed a superotemporal justabulbar GDD, located at the equator of the ten o’clock position. The GDD had a transscleral communication from the plate’s fibrocapsular draining space to the suprachoroidal space (fistula) in the posterior/inferior location, which resulted in a focal pocket of choroidal detachment (Figure 2). The dimensions of the fluid reservoir were 4.97 mm high × 13.66 mm in circumferential diameter × 17.59 mm in antero-posterior diameter, and the dimensions of the pocket of choroidal detachment were 3.91 mm thick × 9.96 mm in circumferential diameter × 11.11 mm in antero-posterior diameter.

**Figure 3:**
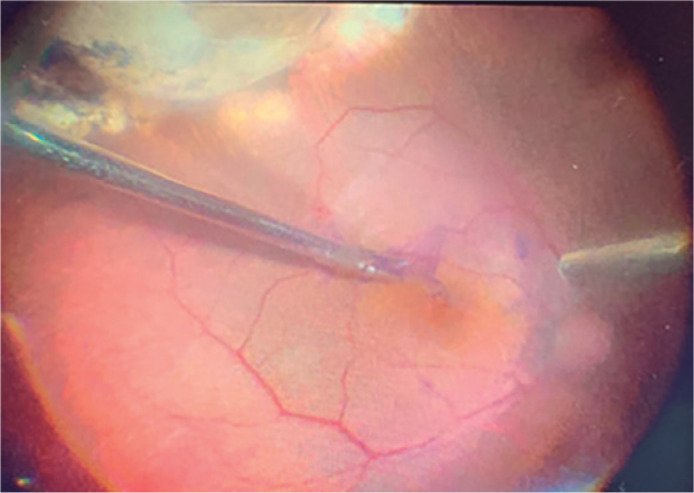
Intraoperative view during the epiretinal membrane peeling showing a localized superotemporal choroidal detachment.

After ruling out a diagnosis of melanoma, we performed a new PPV to remove the epiretinal membrane, internal limiting membrane, and dexamethasone implant as shown in Figure 3.

**Figure 4:**
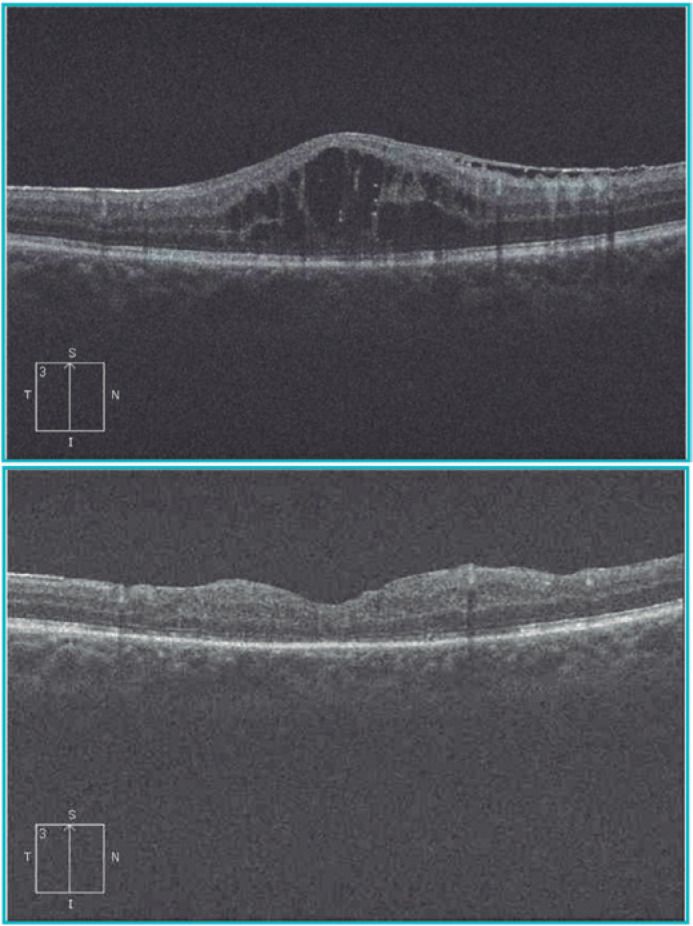
OCT (A) pre- and (B) postsurgical treatment (Cirrus HD OCT, Zeiss, Switzerland). OCT, optical coherence tomography.

The patient was stable, his legion remained unchanged, and his IOP was controlled after surgery. The patient’s visual acuity improved to 20/70 (0.54 logMAR), and we determined that his low visual acuity was caused by the epiretinal membrane and not by the fistula. Figure 4 shows the OCT pre- and postsurgical treatment.

## DISCUSSION

The Ahmed valve GDD is a well-established device for the treatment of refractory glaucoma; however, its implantation can lead to serious complications such as choroidal detachment, a shallow anterior chamber, intraocular hemorrhage, erosion, and retinal detachment^(2,4)^.

Choroidal detachment is one of the most common complications of glaucoma filtering surgeries with an incidence rate ranging from 16.9% to 35.1%^(5)^. A persistent and large choroidal detachment is often associated with significant morbidity, especially when accompanied by other complications such as hypotonic maculopathy^(5,6)^.

We described a case of a patient who developed an atypical choroidal detachment in a distinct location in the superotemporal area after having surgery to implant an Ahmed valve. The patient’s detachment was caused by a transscleral fistula to the GDD’s fibrous capsule, which simulated a choroidal amelanotic melanoma. In this case, we speculated that the fistula was caused by erosion of the scleral wall under the GDD plate, but we could not identify a probable causal factor for the erosion. According to the literature, possible etiologies of erosion include thin scleral walls, surgical trauma, excessive cauterization, continuous trauma to the GDD caused by excessive eye movement, and other unknown factors^(6)^. In the current case, neither excessive cauterization, cryopexy, or surgical trauma were reported. The patient’s scleral wall had an axial length of 23.17 mm, and thus was not considered to be thin associated with high myopia.

Erosions of GDD implants always occur from the implant toward the exterior ocular tissues (the scleral flap ad conjunctiva). We were unable to uncover any previous research describing erosion of the internal scleral wall due to a GDD implant or its associated material. Internal scleral erosion has only been described in conventional retinopexy procedures using a scleral buckle in which the buckle itself exerts pressure on the scleral wall^(6)^.

During the patient’s ultrasound, we observed the presence of a moderate-sized echolucent fluid reservoir, which is indicative of GDD patency^(7)^. We did not observe, however, any flattening of the sclera as this is common only with larger blebs^(7)^.

Choroidal melanoma can simulate choroidal detachment. Kase et al. described a case report of a patient with rhegmatogenous retinal detachment and choroidal melanoma that was initially diagnosed as a choroidal detachment^(8)^. The researchers reported that unless the tumor is medium to large in size, a clinical diagnosis of choroidal melanoma can be mistaken for a choroidal detachment.

Shields et al.^(9)^ have described circumstances that could confuse the diagnosis of choroidal melanoma as well, including choroidal nevus, peripheral exudative hemorrhagic chorioretinopathy, congenital retinal pigment epithelial hypertrophy, idiopathic hemorrhagic retinal detachment, choroidal hemangioma, and age-related macular degeneration. They were able to identify particular cases involving difficult diagnoses in which patients had lesions resembling both choroidal detachment and choroidal melanoma.

In conclusion, the case describes an atypical choroidal detachment simulating an amelanotic melanoma caused by a posterior transscleral fistula from the Ahmed GDD to the suprachoroidal space. To the best of our knowledge, this is the first case described in the literature with these characteristics.
